# Operational State Recognition of a DC Motor Using Edge Artificial Intelligence

**DOI:** 10.3390/s22249658

**Published:** 2022-12-09

**Authors:** Konstantinos Strantzalis, Fotios Gioulekas, Panagiotis Katsaros, Andreas Symeonidis

**Affiliations:** 1 School of Electrical and Computer Engineering, Aristotle University of Thessaloniki, 541 24 Thessaloniki, Greece; 2 5th Regional Health Authority, 411 10 Larissa, Greece; 3 School of Informatics, Aristotle University of Thessaloniki, 541 24 Thessaloniki, Greece

**Keywords:** convolutional neural networks, DC motor failures, EDGE-AI, internet of things, predictive maintenance

## Abstract

Edge artificial intelligence (EDGE-AI) refers to the execution of artificial intelligence algorithms on hardware devices while processing sensor data/signals in order to extract information and identify patterns, without utilizing the cloud. In the field of predictive maintenance for industrial applications, EDGE-AI systems can provide operational state recognition for machines and production chains, almost in real time. This work presents two methodological approaches for the detection of the operational states of a DC motor, based on sound data. Initially, features were extracted using an audio dataset. Two different Convolutional Neural Network (CNN) models were trained for the particular classification problem. These two models are subject to post-training quantization and an appropriate conversion/compression in order to be deployed to microcontroller units (MCUs) through utilizing appropriate software tools. A real-time validation experiment was conducted, including the simulation of a custom stress test environment, to check the deployed models’ performance on the recognition of the engine’s operational states and the response time for the transition between the engine’s states. Finally, the two implementations were compared in terms of classification accuracy, latency, and resource utilization, leading to promising results.

## 1. Introduction

The fast-growing deployment of Internet of Things (IoT) systems to modern industrial environments has led to the development of predictive maintenance applications that implement prognostic algorithms to monitor the “health” conditions of an asset in order to minimize the operational costs and ensure safety. In this context, data processing along with cloud-based analytics are used to detect possible mechanical failures and propose corresponding maintenance tasks. This approach is efficient in terms of accuracy; however, time delays in data transfer, possible bandwidth saturation, or network outages render it prohibitive in applications where hard or soft real time constraints are imposed (e.g., industrial environments). Thus, there is a need to shift the time delay associated with computations to the local layer by integrating intelligence into devices at the edge of systems. Most IoT devices provide limited resources in computing power and memory and usually generate large amounts of data; therefore, through integrating intelligence to them, we also reduce the cloud processing overload. This implies supporting a kind of “consciousness” to the devices that become capable to interact with or even without an internet connection and can be immediately adapted to rapid state changes [1].

Edge computing is economically advantageous and easier to access when compared to cloud computing and can support a wide variety of artificial intelligence (AI) applications. Therefore, edge computing is an important avenue in the evolution of AI and its applicability to predictive maintenance [2]. AI has the potential to simplify the design of the complex processing involved in such applications and can be used also to control and classify the processes of a production line by learning features directly from the extracted data. In effect, the “health” control of a factory machine system and its operational conditions enables the associated operator to anticipate and schedule the required maintenance, thus allowing the predictive maintenance prospect to extend the operation time and life of industrial machines and eventually save costs. By moving AI computations as close as possible to the sensor (EDGE-AI), the scalability and responsiveness of the system can be improved and the real-time requirements that are usually seen in industrial environments can be met [3].

In this paper, we focus on the timely and effective operational state recognition of a direct current (DC) motor, which is an essential element of practical interest in industrial environments. In this domain, motors are inspected on a regular basis to ensure their efficient operation. The main causes of faults in electrical motors include electric overload, low resistance, overheating, dirt, moisture, and vibrations [4,5,6]. The maintenance plan is performed through the identification of the causes of the aforementioned failures and the execution of proper actions to mitigate them or to lower their impact. The occurrence of operational problems should, therefore, be resolved in short timeframes to avoid further failures. Within this scope, possible motor failures have to be detected on time, since this is crucial to avoid adverse effects during the operation of the overall setup and instrumentation. 

By eliminating the need of data transfer to the cloud, EDGE-AI can increase the inference speed of a motor’s operational state recognition. A direct consequence, in terms of the motor’s operation, is the improved safety and reduced maintenance costs. In terms of reliability of the maintenance system, potential problems due to poor network connectivity can be avoided, since no internet connection is required.

Within the outlined context, we present the design and implementation of a convolutional neural network (CNN) on a micro-controller board that supports digital omnidirectional microphones to sense the sound and infer the operational states of a DC motor. Two methodological approaches are implemented, each of them based on a different open-source EDGE-AI tool. The experimental results demonstrate the applicability of the proposed EDGE-AI solutions to the timely and effective operational state recognition of a DC motor for the purpose of its predictive maintenance.

In summary, the contributions of this article towards the implementation of EDGE-AI nodes for operational state recognition of DC motors are:

- An architecture design of the EDGE-AI implementation and the frequency analysis of an audio dataset [7] for a DC motor’s operational states. We show, in detail, how the sound signal is digitally processed and interfaced with the deep learning (DL) algorithm.
- Two design methodologies for the implementation of CNNs on microcontroller units (MCUs). We evaluate the utilization of HW resources, along with the model training and validation processes.
- The simulation of a stress test experimental setup, which included slight variations in the environmental noise gain, so as to evaluate the performance of our EDGE-AI solutions in conditions close to a real industrial environment. More concretely, the inference performance is measured with respect to the resulting speed and accuracy, two criteria crucial for the stringent real-time conditions of an industrial environment.

To the best of our knowledge, this the first piece of work to be reported in the literature that presents and analyzes, in detail, the implementation of CNNs on MCUs (two methodologies), for the operational state recognition of DC Motors, based on sound signal processing and classification. It is shown that our implementation is suitable for EDGE-AI classification, which can be incorporated into a predictive maintenance process for industrial environments.

The rest of the paper is organized as follows. Section 2 reviews the related work in the field of DC motors’ predictive maintenance and the incorporation of EDGE-AI features. Section 3 gives an overview of the characteristics of the dataset used for the training and testing of the deep learning models and introduces the necessary definitions for sound signal processing and the key components for the real-time execution of these models on the edge. Section 4 presents the architecture of the EDGE-AI node and the flow of data operations. Section 5 describes the two design methodologies and evaluates their performance in various environmental sound conditions in terms of the real-time classification capabilities of the generated CNN models. Section 6 compares the EDGE AI implementation against a cloud computing solution. Finally, Section 7 concludes the paper and provides insights on future research directions.

## 2. Related Work

A recent analysis on the adoption of IoT technology [8] reported that a share of 29% of the industry has been invested in the deployment of real-time IoT sensor data and AI techniques to determine when maintenance should be performed on specific equipment. As a consequence of this trend, predictive maintenance has enabled the active monitoring of highly critical assets by aggregating and analyzing in real time multiple sources of data, such as temperature, humidity, sound, vibration, and noise. Within this context, several methods of maintenance have been proposed for the diagnosis of DC Motor faults and the state of their operation conditions. Table 1 summarizes the main characteristics of the methods presented in the literature. The shown methods are classified based on the monitoring variables of the DC motor operation, the employed diagnostic algorithm and the use or absence of cloud support.

Specifically, authors in [9] presented an approach for fault recognition in induction motors using a low-cost Arduino-based sensor system for control. Their method utilized a fault recognition system, a speed control system, and a data collection system. In another approach, Ref. [10] presented a fault diagnosis method for DC motors at the early stage based on machine learning models. A dual-tree complex wavelet packet transform was applied to extract the features of current measurements and a support vector machine (SVM) classifier was used to diagnose the faults. As a second approach, a CNN was used for classification, while in another variant a combination of CNN and recurrent neural network (RNN) was employed to classify the faults. The final decision upon the adoption of the model was dependent on the signal-to-noise ratio (SNR) value. Additionally, authors in [11] addressed the problem of the fault tolerant control of a DC motor, based on a cloud-assisted system. With this type of control strategy, they combined network resources and computing resources to reduce the cost of various control tasks.

In [12], the fault diagnosis of DC motors was based on the implementation of decision trees for the analysis of audio signals. For the experimental evaluation, defects were created using electrical discharge machining (EDM) to keep the size of the defects under control. Furthermore, authors in [13] presented a similar technique, according to which the diagnosis was composed of two processes: the pattern creation process and the pattern recognition process. In the analysis, acoustic signals from the DC motor were used to identify the motor’s operational conditions. The motor states considered were: a “Healthy” DC motor, a DC motor with six short-circuited rotor coils, and a DC motor with a damaged coil and six short-circuited rotor coils.

In another methodology, the authors of [14] described a method of bearing fault detection using widely accessible audio tools. In the experiments, audio measurements from a smartphone and a USB microphone, as well as vibration measurements from an accelerometer were collected for an induction motor, showing a number of bearing irregularities. The faults were classified using a Matlab routine for executing the SVM analysis [15]. In [16], the authors present a sound-based fault diagnosis technique where five engine states were identified: healthy motor, motor with damaged rotor coil, motor with short-circuited stator coil, motor with a broken tooth in the gear, and motor with damaged gear. The following methods were used to classify the engine conditions from the extracted features: nearest neighbor (NN), nearest mean (NM), self-organizing map (SOM), and backpropagation neural network (BNN). The proposed technique using a one-dimensional convolutional neural network (1-DCNN) in [17] had the limitation that if the engine operated quietly, it was difficult to get good results. In [18], a method of fault detection in motors with rotating elements based on audio signals was proposed. The classification process was performed with an artificial neural network (ANN) using back propagation.

The related work also includes proposals for the deployment of EDGE-AI on microcontrollers for predictive maintenance and sound-based classification. The experimental setup that is described in [3] shows how AI can be used effectively to recognize and classify loads in a drivetrain using an STM32 microcontroller. For this purpose, an accelerometer was used to measure the vibrations of the system. In addition, the same study illustrates how DNNs are constructed and trained for classification purposes and how the MCU code library and the compressed AI model are then automatically created with the X-CUBE-AI tool [18]. The training of the DNN model was performed using the Keras Python library [19]. Additionally, in [20], an audio classification method based on a microcontroller is also demonstrated. This application focuses on classifying sound signals produced by car engines in order to manage traffic in urban centers. The methodology used ANNs running on a microcontroller with a microphone, without need for a durable connection to a server. The application was executed on the STM Sensortile Kit board with 128 KB RAM and 1 MB flash memory [21]. First, the MFCCs (mel-frequency cepstral coefficients) were extracted for audio pre-processing. The classification of audio events was based on a pre-trained convolutional recurrent neural network (CRNN).

Taking into consideration the aforementioned approaches, this work addresses two major challenges: (i) Is it possible to identify the operational states of a DC motor using only acoustic signals without attaching extra sensors to it (e.g., accelerometers or gyroscopes to capture vibrations and changes on the positions of the moving parts, stator current measurements)? (ii) Is it possible to implement an AI algorithm on a resource-constrained MCU that is interfaced to a microphone to perform the inferences without sending data to the cloud? Within this context, the proposed work focuses on the implementation of AI on a resource-constrained MCU to perform sound classification in order to identify the operational states of a DC motor and diagnose faulty conditions in real time. In comparison to the related work, we define the architecture of the signal-processing path required to transform and feed the audio signals, captured from the on-board microphone to the CNN. Additionally, the design methodologies used in both industry and academia for the CNN implementation on MCUs are analyzed and assessed. Furthermore, EDGE-AI and cloud computing modes are evaluated in terms of latency and data transfer. To this end, we demonstrate how this EDGE-AI approach can help to optimize predictive maintenance processes by increasing efficiency and reducing costs in the business operational flows of industrial environments.

## 3. Methods and Materials

### 3.1. DC Motor Dataset

The proposed technique and its implementation were tested and validated using the IDMT-ISA-ELECTRIC-ENGINE dataset [7] from Fraunhofer Institute for Digital Media Technology (IDMT). This dataset consists of audio recordings from a DC motor for three operational states of the motor (“good”, “broken”, “heavy load”). The dataset was generated using the ACT Motor Brushless DC 42BLF01, 4000 RPM, 24VDC. The measurements were taken through an improvised microphone with the following parameters: frequency range 50 Hz to 20 kHz, voltage range 2 V to 10 V, omnidirectional, sensitivity −35 dB ± 4 dB. Additionally, to record the data we used 44,100 Hz sampling frequency, 32-bit resolution, mono audio, and WAVE format.

For the audio signals recording, three identical motor units were employed to simulate different sound situations. The first motor operated at 60% of the supply voltage and represented the “good” operating state. For the second motor, the supply voltage varied every 18 ms between 15% and 75% of the supply voltage to represent the “broken” operating state. The “heavy load” case was set when an additional load was applied to the third motor, with a supply voltage of 60% of the nominal. The dataset consists of the audio recordings of the electric DC motor in combination with the presence of the following types of sound environments:

- Pure—recordings without the presence of other sounds or noise.
- Talking—recordings with the presence of sounds from people talking outside the case surrounding the device.
- White noise—recordings in the presence of white noise played by speakers outside the case surrounding the device.
- Atmo—recordings with the presence of sounds from a factory environment in three volume levels (low, medium, high) that were reproduced using speakers.
- Stress test—recordings with slightly changed gains at the input, simulating variations in the setup.

**Figure 1:**
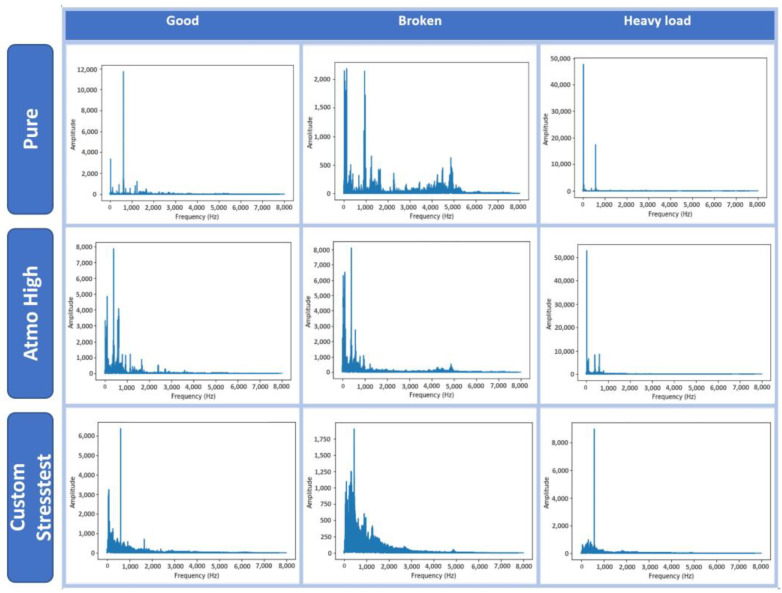
Frequency responses of audio signals.

Table 2 lists the audio recordings with their type and duration included in the IDMT-ISA-ELECTRIC-ENGINE dataset. All recordings are equally divided into three classes (good, broken, heavy load) in terms of their duration. The duration of each individual sample is 30 s. The frequency response is shown in Figure 1.

### 3.2. Basic Definitions

Henceforward, we provide the basic definitions for the terminology and the concepts used throughout the next sections of the paper.

#### 3.2.1. Mel-Scale

Log-mel spectrograms and the associated MFCCs are used extensively in deep learning frameworks for various tasks, such as emotion recognition, audio classification, and automatic speech recognition (ASR) [21]. Log-mel spectrograms are produced by applying the mel-scale transform of the sound data and afterwards the logarithmic scale conversion. The MFCCs correspond to the coefficients that define a mel-frequency cepstrum representation of the short-term power spectrum of a sound, based on a linear cosine transform of a log power spectrum on a nonlinear mel-scale of frequency. Furthermore, the conversion to mel-scale is the result of the nonlinear transformation of the frequency f (Hz) into m (Mels), as shown in Equation (1) [22].

(1) m=2595log(1+f/700),

#### 3.2.2. Convolutional Neural Networks (CNN)

A CNN consists of a set of nodes organized in multiple layers by stacking many hidden layers on top of each other in sequence, which receive inputs from a previous layer and compute an output from a weighted and biased sum of the inputs in a feed-forward and hierarchical manner. During training, data is introduced to the input layer of the network, and the output of each layer is sent to the next one. The last or the so-called output layer yields the model’s predictions, which are compared to the known expected values to evaluate the model error. The training process involves refining or adjusting the weights and biases of each layer of the network at each iteration using a process called back-propagation, until the output of the network closely correlates with the expected values. Therefore, the network iteratively learns from the input dataset and progressively improves the accuracy of the output prediction.

#### 3.2.3. Softmax

The softmax function is a generalization of the logistic function to multiple dimensions and is applied to multinomial logistic regression or performed during the last activation function of a neural network, where it normalizes the output of a network to a probability distribution over predicted output classes. The standard (unit) softmax function $\sigma :\;{\mathbb{R}}^{k}\to {\left[0,1\right]}^{k}$ is defined in Equation (2) [23]:

(2) $$\sigma {\left(z\right)}_{i}=\frac{e^{z_{i}}}{\sum_{j=1}^{k}e^{z_{j}}}$$

for i=1, …, k and $z=\left(z_{1},\ldots ,\;z_{k}\right)\in {\mathbb{R}}^{k}$.

#### 3.2.4. Rectified Linear Unit (ReLU)

In the context of artificial neural networks, the rectifier or ReLU activation function is an activation function defined as the positive part of its argument f(x)=max(0,x), where x is the input to a neuron [23].

### 3.3. Design Methodology for Artificial Intelligence Implementation on Edge and IoT Devices

**Figure 2:**
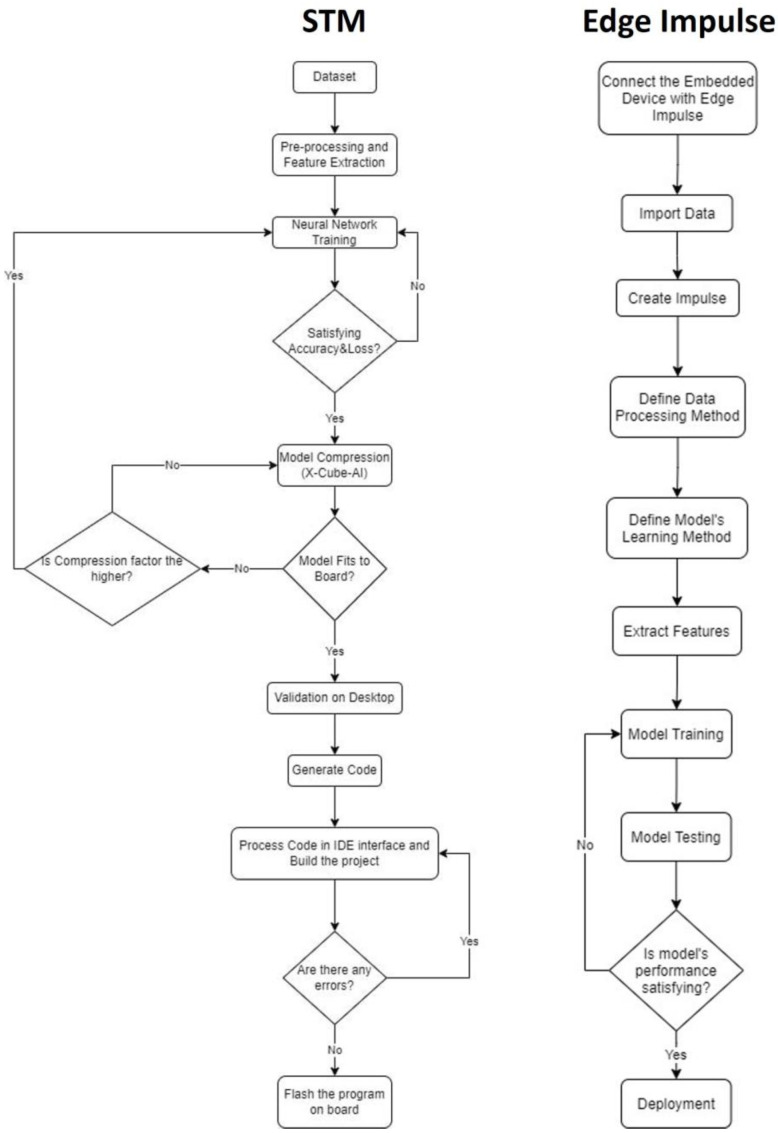
Design flow variants based on the STM software tools and the Edge Impulse platform.

We followed two variants of a design flow using appropriate environments for the integration of a neural network into an MCU, i.e., the STM software tools [18] and the Edge Impulse platform [24]. The implemented models were trained and evaluated using the sound dataset mentioned before [7]. Figure 2 depicts the steps of the two aforementioned approaches towards the AI models’ implementations on the STM32 Discovery Kit IoT node board [25] in order to present a high-level description regarding the different processes during the progression of the entire development for each technique. The board featured an ARM Cortex-M4 CPU with 64-Mbit Flash memory, Bluetooth, Wi-Fi and LoRa connectivity, Dynamic NFC tag, two digital omni-directional microphones, and various peripherals and sensors. The implementation based on STM’s tools included pre-processing, feature extraction, and data labelling of the sound dataset, which were eventually fed to the selected neural network for training and evaluation of its performance. The successful completion of the training process was determined by the accuracy, as well as the loss function.

The accuracy was required to be as high as possible at the end of the training process, following a smooth progression over the epochs. On the other hand, the loss had to be as low as possible at the end of the training process, following a smooth reduction over the epochs. At the end of the neural network training process, we extracted the trained model in order to import it to the STM’s CubeMX software tool [18]. The model can be inserted in several forms, such as .h5, but it is preferable to be extracted in the most compressed form possible, especially if it is a large network, because it will be imported after further compression into a microcontroller device with limited memory capacity. Therefore, the trained model was transformed to a TensorFlowLite format. This conversion was based on a post-training quantization. Such a process focuses on the reduction in the trained model’s size and the improvement at the CPU level towards increasing the inference speed. Subsequently, for the model’s compression stage through CubeMX, it was necessary, firstly, to select the board on which the project will run on. Then, we selected the X-Cube-AI as an additional software tool. This particular software tool was able to convert a trained model in a form that could run on an STM microcontroller. After the completion of this step, we imported the trained model into X-Cube-AI. It was then necessary to analyze the model through the software tool to ascertain whether it fitted to the selected microcontroller. The analysis outputs included the RAM and flash memory occupation of the model, as well as the multiply and accumulate (MACC) number, which represents the number of operations to perform the inference. If the RAM and/or flash memory occupation of the model exceeded the board’s limits, there was a compression choice available. If the compression failed to drop the memory occupation enough so that the model can fit to the selected board, then post-training quantization would be needed, as we already mentioned. If the post-training quantization and the extra compression provided by X-Cube-AI was not adequate, then the topology of the neural network would need to be changed in a way to allow the model to properly predict its classes. The next step was the model’s validation on the desktop by using either random numbers or by importing a test set from the dataset on which the model had been trained on. The validation on desktop stage performed a comparison of the original model trained in Python and the compressed model produced by the X-Cube-AI tool, so that the user can realize whether they converge or not. After the validation on desktop, it was necessary to configure the application by going to platform settings and selecting the appropriate COM port. Moreover, it was important to configure the Pinouts and the clock according to the peripherals to be used via the application that will run on the board. Finally, we needed to generate the C-code project by selecting the preferred Toolchain/IDE. The generated code contained the topology of the neural network, as well as other key functions for the application functionality. The final stage involved programming all the necessary functions appropriately, debugging, and flashing the executable program on the board.

Edge Impulse [24] is a platform that can be used for AI projects on embedded devices. The methodology for deploying a project end extracting the executable program was simple. Initially, we needed to connect the board to be used with the Edge Impulse environment. Next, we imported the data to be used for the training part, as well as for validation and testing. The procedure took place either by importing the data files from a PC or by collecting the data directly from the board’s sensors. When importing the data, we also needed to label them according to the classes of the AI project. Then, through the “Create Impulse” tab, an impulse was created that takes raw data, uses signal processing to extract features, and then employs a learning block to classify new data. When the signal processing method (e.g., MFCCs) and the learning block (e.g., neural network) are chosen, it is necessary for them to be configured according to their parameters. Edge Impulse provides a proposed parameter setup for each method. After setting all parameters for the feature extraction, as well as the learning method, the model was ready for training. At the end of the training process, Edge Impulse displayed a panel where the model’s performance was depicted. Moreover, a “Live Classification” option was provided, whereby connecting the board to be used, Edge Impulse could capture data from its sensors and could also classify them based on the trained model. Through the “Model Testing” tab, test data could be classified, and the user could thus decide if the model is efficient enough. The final phase was the deployment of the model (“Deployment” tab), which involved the selection of the inference engine and the output format. The binary file could be extracted directly from Edge Impulse for a variety of boards.

The main differences of the two methods are summarized as follows: (i) although the STM tools supports only the automated conversion of a trained model into an equivalent C model, it requires that the entire pre-processing, feature extraction, and model’s training have to be performed manually and through iterations (ii) Contrarywise, Edge Impulse platform provides a more automated solution, where the engineer just pre-configures all the associated parameters for the entire process, taking into account the problem’s domain (in our case sound processing and deep learning). (iii) An EDGE-AI implementation based on the STM approach provides more degrees of freedom for the developer but it requires full expertise in machine learning/deep learning and embedded systems, while Edge Impulse exhibits a more user-friendly approach, albeit more constrained in development interventions by the user.

## 4. EDGE-AI Node Architecture and Operating Process

**Figure 3:**
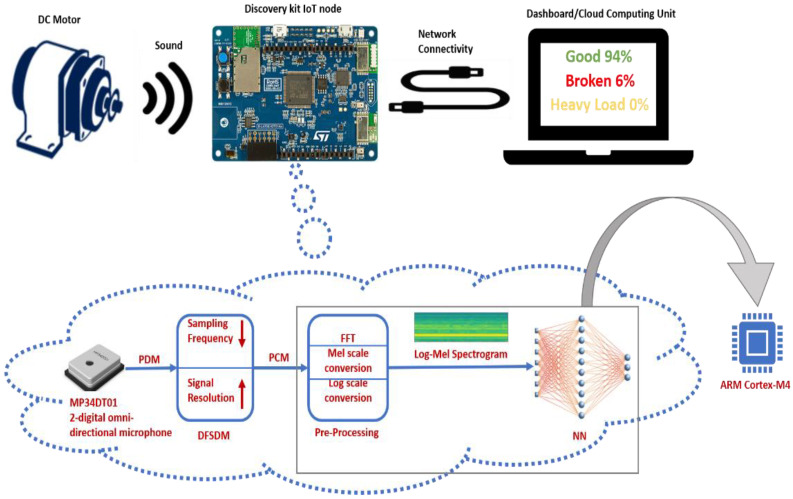
Architecture and block diagram for the DC motor’s operational state recognition via the EDGE-AI node.

Figure 3 depicts the block diagram of the DC motor’s operational state recognition via the utilization of the EDGE-AI node, along with the data flow procedures from audio signal reception until its processing by the neural network that runs on the board and the generation of the inference. The sampling frequency of the digital omnidirectional microphones (i.e., MP34DT01 module) of the STM32 IoT node [25] is 16 kHz (16 bit, 1 channel), which means that the acquired digital signal is represented in PDM (pulse-density modulation) format. Therefore, through the peripheral DFSDM (digital filter for sigma-delta modulators) that handles the sound of the microphones, the PDM samples are collected and converted to PCM using the peripheral configurations, where the output sampling frequency is set to 16 kHz and the output signal resolution at 16 bits. The PCM samples are then stacked in a 1024 sample rolling window with 50% overlap. Every millisecond, a DMA (direct memory access)-based interrupt is received with the last 16 PCM (pulse-code modulation) audio samples. The DMA controller supports the transfer of data from peripherals and memories without being loaded into the CPU. For every 512 samples (i.e., 32 ms), the buffer is inserted into the pre-processing process to extract the attributes. The pre-processing process outputs audio features to a LogMel spectrogram (30 × 32). For computational efficiency and optimization in memory management, this processing step is divided into two parts. The first part calculates one of the thirty-two columns of the spectrogram from the time domain for the input signal on the mel scale, using the fast Fourier transform (FFT) and the application of 30 mel filters. In the second part, when all 32 columns of the spectrogram are calculated (after 1024 ms), a log-scale conversion is applied to the Mel-scale spectrogram, thus shaping the input features for the neural network that runs on the MCU. Every 1024 ms, the (30 × 32) LogMel spectrogram is fed to the input of the neural network, which, in turn, classifies it into the outputs: “good”, “broken”, and “heavy load”.

## 5. Methodological Approaches—Results

### 5.1. EDGE-AI Implementation Based on STM’s Tools

The Python programming language was used for pre-processing the audio data from the audio dataset [7], feature extraction, and model’s training process. The STM32CubeMX tool and its extension, X-CUBE-AI [18], were then used to convert the neural network into a format that allows it to be added and operated in the STM32 IoT node [25]. The Python’s Librosa library [26] was utilized for data pre-processing. Initially the audio samples were subject to resampling from 44.1 kHz to 16 kHz. The data was then divided into smaller subsections for the creation of 32-column LogMel spectrograms. Subsequently, FFT was applied on sections for the conversion from the time domain to the frequency domain. The length of FFT was 1024 samples, with the hop length [26] being 512 samples. Next, data was converted to Mel scale by applying 30 triangular overlapping filters, and consequently were converted into LogMel spectrograms of 30 × 32 size. These features were used to train, validate, and test the model after being normalized with Z-score normalization. The particular normalization involves redefining features so that they have the properties of a standard normal distribution with an average of zero and a standard deviation of one [27,28]. During the training process, the accuracy and loss of the model on the data were evaluated. Therefore, the dataset was separated into training and validation sets to assess the accuracy and loss at the end of each training session. The testing set was only used for the final evaluation as unseen data. Specifically, 25% of the total dataset for the testing set was randomly selected. Then, from the remaining 75%, 25% was again randomly selected for the validation set, while the remaining dataset represented the training set.

We used a sequential model, implemented in Keras [19], to build the CNN model layer by layer. The CNN consists of two convolutional layers (Conv2D), two max pooling 2D layers, one flatten layer, and two dense layers. Specifically, the first convolutional layer was placed, which received a LogMel spectrogram as input as a two-dimensional matrix 30 × 32 in the form (30, 32, 1) (l × m × r). The number of filters was 16 with a size of 3 × 3 (n × n). In general, in CNNs the number of filters is smaller than the dimension of the input data. This option was preferred because it allowed the same filter (weight set) to be multiplied by the input panel multiple times at different input points. As a result of the first layer, 16 feature maps of size (l − n + 1) × (m − n +1) were produced, i.e., 28 × 30. ReLU was selected as the activation function. Each feature was then sampled using a max pooling layer in 2 × 2 continuous areas, maintaining the maximum value from each area. As a result, the out was reduced to 14 × 15.

The second convolutional layer had the same 16 filters of size 3 × 3, and ReLU activation function. As before, there was a max pooling layer after the convolutional layer with sampling in 2 × 2 continuous areas. The output of the second convolutional layer was 12 × 13, while the output of the second max pooling layer was 6 × 6. After the second max pooling layer, a flatten layer follows, which results in the consolidation into one column of size 576. Finally, two dense layers were used, the first with nine units and ReLU activation function, and the second with three units and a softmax activation function. The choice of softmax was made, as in the case of the second layer, due to the multiclass classification nature of our problem, where the three units represented the three classes (i.e., “good”, “broken”, and “heavy load”). The model’s output constitutes a probabilistic classification for every spectrogram fed in the CNN, regarding the three classes (i.e., three percentage indications, one for each of the class). The data for training and validation was set with batch sizes equal to 500, and 10 epochs were used for the training process, due to the fast convergence that was observed at a high accuracy value, as well as at a low loss value. Specifically, after the 10th epoch, a training accuracy of 99.91% and a validation accuracy of 99.87% were achieved. Regarding the loss, a training loss of 0.0073 and a validation loss of 0.0074 were achieved. For the confusion matrix shown in Table 3, 31,902 testing features were used that correspond to 25% of the complete dataset.

In order to deploy the trained model into the STM32 Discovery kit IoT node, it must be saved in a format so that the X-CUBE-AI tool can create the corresponding optimized C model for the specific STM32 device. The model was initially saved in .h5 format (HDF5 file), but for further optimization a conversion to TensorFlowLite model was performed. This transformation reduced the size of the model while it also reduced the transfer delay to the CPU and improved the hardware acceleration at a small cost in terms of model accuracy. Specifically, it was chosen to apply integer quantization, an appropriate choice for the use of models in MCUs. This process essentially converts 32-bit floating-point numbers, such as weights and activation outputs, to the nearest 8-bit fixed-point numbers. The end result was a .tflite format.

**Figure 4:**
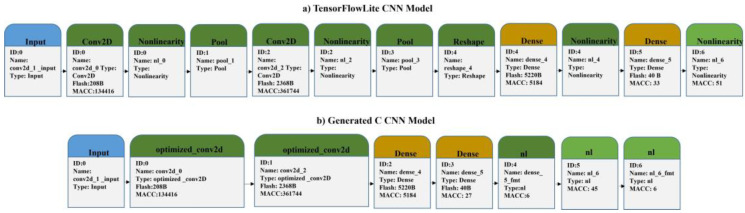
CNN model in TensorFlowLite and generated C-Model from STM tools.

After the completion of the previous procedures, the produced TensorFlowLite model was inserted into the X-Cube-AI tool of the CubeMX environment. The tool performed the weight compression of the CNN, the merging of network layers optimization regarding the utilization of RAM and ROM, and the generation of a C-based model of the CNN, which comprises all the necessary files for the topology, weights, and bias of the CNN. The complexity of the C-model was 501428 MACC and the utilization of the flash and RAM memory was 7.65 Kbytes and 5.52 Kbytes, respectively. After extracting the necessary libraries to produce the project in the CubeIDE environment for the final deployment stage, there was a validation process that was performed on the board by importing data from the testing dataset used for the corresponding procedure in Python. After the model’s analysis completion in X-CUBE-AI, there were two network forms (i.e., the original TensorFlowLite model and the generated C model), as illustrated in Figure 4. Specifically, the boxes representing the layers also contain information, such as flash memory consumption and the number of MACC operations. As a visual effect, it can be observed that in the created neural network (Figure 4), the convolutional layers are joined with those of the pooling, as well as those of the nonlinear ones (activation functions). Furthermore, the generated CNN model’s architecture has been differentiated from the initial model.

It was observed that the largest percentage of the execution time was concentrated in the optimized convolutional layers and that the total execution time was 40.767 ms, which constitutes a very satisfactory result. Moreover, the C-model showed a very good fit to the control data; as 100% accuracy was obtained, root mean square error (RMSE) was only 0.001059 and medium average error (MAE) was 0.000112, where imported network’s forecasts were taken as reference values for the comparison. Finally, the confusion matrix (using 1901 samples) for the control process based on the C-Model is presented in Table 4.

### 5.2. EDGE-AI Implementation Based on Edge Impulse Environment

A second implementation was based on Edge Impulse environment that facilitates a more automated development process. This environment does not require an in-depth intervention by the designer during the entire development procedure. After importing the audio files into Edge Impulse, the data was split to 1 s windows with a 0.5 s overlap. For feature extraction, the mel frequency cepstral coefficients (MFCC’s) option for audio pre-processing was chosen. Moreover, FFT was performed with a length of 256 samples with 20 ms frame length. Subsequently, 32 mel filters and 13 cepstral coefficients were selected. Another important step for the development of the methodological approach based on the Edge Impulse environment was the development and training of a CNN model with a different architecture, in comparison with the model that was developed in the first methodological approach (i.e., the approach based on the STM tools).

Specifically, the input (i.e., MFCC spectrograms) feeds the first layer of the CNN. The spectrograms were converted during their passage through the intermediate layers into three numbers that represented the probabilities representing each sample’s classification to “good”, “broken”, and “heavy load” class. For the intermediate layers, initially, a reshape layer was placed. The first convolutional layer had one-dimensional convolution window length (Conv1D); it included 30 filters, kernel size 5, and activation function ReLU. Next, there was a max pooling layer, with pool_size = 5. Subsequently, the second convolutional layer (Conv1D) was placed, which had 10 filters and the same other parameters as the first convolutional layer. A max pooling layer followed, configured with the same parameters as the first max pooling layer. After the second max polling layer, a flatten layer was placed and finally a dense layer with softmax as activation function. Adam was used as an optimizer, while the categorical cross entropy function was chosen as a loss function and the accuracy metric for the model’s performance evaluation. Finally, 90 epochs were used for the training process of the model.

After having selected the STM32 Discovery Kit IoT Node board from the platform, it was possible to choose between two options for the CNN’s deployment, the quantized and the non-optimized model. The quantized model was selected because it presented better performance in memory levels (RAM and ROM), as well as in response time, compared to the non-optimized model. The accuracy of the quantized model was 93.3%, while the corresponding one for the non-optimized model was 93.38% (negligible difference). The Edge Impulse automatically produced the .bin executable file that could be executed directly on the board.

The results from the CNN model’s training in the Edge Impulse environment for 90 epochs, in terms of accuracy and loss, are provided in Table 5, which also illustrates information regarding the inference time, maximum RAM, and ROM usage for the model. It is noted that the performance of the model was calculated based on the characteristics of the STM32 Discovery Kit IoT node (Cortex-M4) and the compiler Edge Impulse EON. The confusion matrix on the control data is listed in Table 6. We observe that the model has high accuracy and a low loss. Additionally, the inference time is very satisfactory, as well as the consumption in RAM and ROM. The performance of the model in the testing data is also very good, as is shown in the confusion matrix.

### 5.3. Real-Time Performance Monitoring and Test Results

The test environment included a set of digital speakers, where we played the recordings created by joining audio samples from the dataset [7] that were not used in models’ training processes. Therefore, for each model, the audio clips were played from the speakers that were placed on either side of the STM32 Discovery Kit IoT Node while, at the same time, each model was executed on the board. Finally, the results regarding the classification performance of the models, as well as their response time regarding the transition of the DC motor from one state to another, were analyzed. Three cases were simulated to calculate the response time of the two implemented CNN models capturing DC motor’s state alternations for each one of the surrounding environment assumptions (pure, atmo High, atmo Medium, atmo Low, talking, white noise, and stress test). These cases were: (i) good-to-broken transition, (ii) good to heavy load transition, (iii) heavy load to broken transition. In addition to the operating environment assumptions provided by the dataset, a custom stress test was simulated during which pure recordings were reproduced, for each of the above three cases of the engine’s state change, with the simultaneous presence of noise generated at the site of the experiment (e.g., speech, impulsive strong noises, music). We analyzed the test results by defining representations of the probabilities for the predictions of the implemented CNN models per sample recording in order to give a visual demonstration for all the simulated environmental assumptions. 

**Figure 5:**
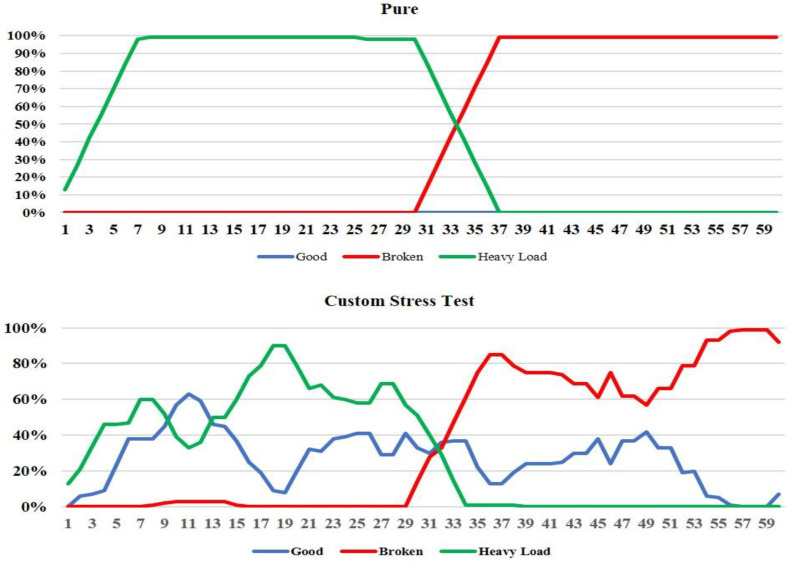
Performance of CNN model of the STM’s methodology for the heavy load to broken state transition test.

**Figure 6:**
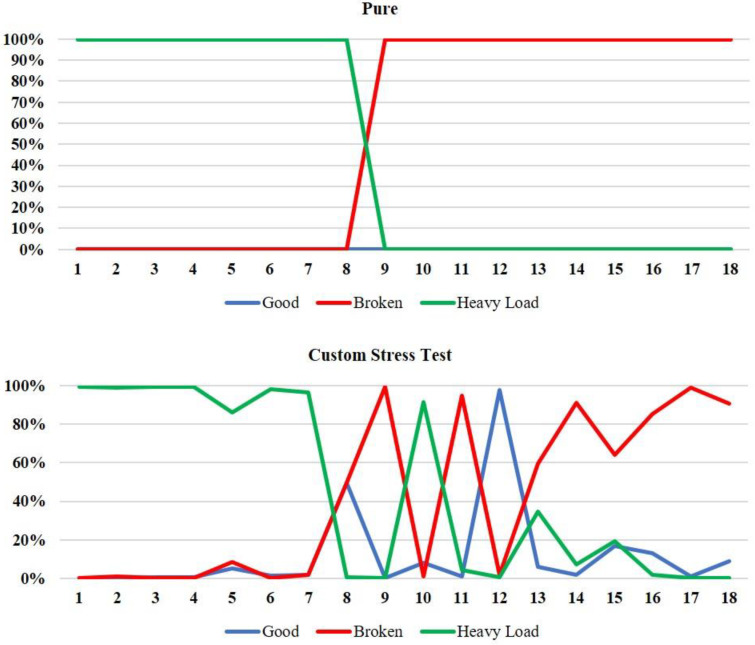
Performance of CNN model of the Edge Impulse’s methodology for the heavy load to broken state transition test.

The diagrams of the recording results of the measurements for the recognition of the DC motor’s operating states consist mostly of 60 measurements for the STM-based CNN model and 18 measurements for the Edge Impulse-based CNN model. This difference in the number of measurements taken between the two models was due to the fact that the STM model performed continuous sampling with the processed samples entering the CNN every 1024 ms for an inference time of 40.767 ms, while for the Edge Impulse model there was a time interval of 2 secs for starting each measurement, 1 sec sampling duration, 16 ms latency, 337 ms for DSP calculations, and 36 ms for classification time. Indicatively, Figure 5 and Figure 6 illustrate the live classification’s performance of the two CNN models, where the horizontal axis represents the audio samples and the vertical axis represents the percentage indications for each of the three classes and for each sample.

The STM CNN model’s performance in the presence of the pure environment was satisfactory and accurate for all of the class transition experiments. As for the custom stress test environmental background, it is, firstly, noted that for the heavy load to broken transition experiment, the model performed well for the broken class, but there was a relatively lower probability of classification on average for the heavy load class, which nevertheless maintains the reliability of the model (>50%). On the other hand, the Edge Impulse model exhibited an accurate classification capability in the presence of the pure background environment for every class transition scenario. Contrariwise, at the custom stress test background environment, there were many fluctuations within the course of the prediction percentages over the audio samples, while the model presented a few misclassifications. Therefore, in the presence of severe noise, there is a possibility that good and heavy load classes exhibit the same signal levels and may cause further delay in the CNN model to converge. This was noticeable in Figure 6 where the EDGE-AI was tested under stress test conditions.

Furthermore, Table 7 and Table 8 summarize the test results for the two models by providing the average percentage indications of each one of them for all measurements and environmental backgrounds during playback of each class, as well as the transition response times, regarding the transition from one class to another. In Table 7, we observe that the STM model performed sufficiently on the recognition of every class, as well as on the response time between the transitions from one class to another. Specifically, it is worth noting that the only difficulty observed for the STM model’s prediction success was for the broken class at the custom stress test environment during the good to broken transition experiment, because it presented an average percentage of 61%. Nevertheless, these slight difficulties of the model for these environments are justified because the background noise was intense. As for the response time of the model between the class transitions, it was found sufficient because most perceptions of the transition took place in under 5 s. On the other hand, Table 8 shows that generally, the Edge Impulse model was capable of correctly classifying every class in all the rest of the environmental backgrounds, except for the stress test environment during the good to broken transition, where it exhibited an average percentage of 54.9%. As for the response time of the model to observe a transition between two classes, in most of the cases, the model needed less than 4 s to recognize a change between the DC motor’s operational states.

### 5.4. Models’ Performance Analysis and Evaluation of Test Results

The performance comparison between the two models (STM and Edge Impulse) was based on the set of results obtained from the experimental tests during their execution on the STM32 Discovery Kit IoT Node, the differences of the two implementations regarding the resources’ utilization, and the capability of developing an integrated application. It is noted that both models demonstrated the ability to adequately detect the various engine operating states for the full range of experiments performed, in terms of average classification success rates (>50% in all cases) and response times regarding the transition between operational conditions, which makes them both capable to be used for the valid and timely detection of the DC motor’s three states (i.e., good, broken, and heavy load).

For each implementation, we derived the corresponding confusion matrices for the whole range of experiments by incorporating all the measurements taken during the reproduction of the audio samples for every operational state separately. For instance, in the case of good operational state, all the measurements collected during the reproduction of this class in the two simulated transition modes containing it (good–broken, good–heavy load) were collected separately for each one of the two models. Subsequently, from all these measurements collected for this class, the number of measurements was separated according to the class identified by the model. This procedure was performed to check the efficiency of the model over the entire range of the experiments performed. It is observed from Table 9 and Table 10 that the STM model infallibly identified the good operational state contrarywise of the Edge Impulse model, which presented a misclassification rate of 10.94% for this class. Regarding the other two classes (i.e., broken and heavy load), it is observed that the Edge Impulse model had a slightly higher accuracy, compared to the STM model.

Furthermore, by checking the average transition response times between modes for both models we observe that the STM model has an average time transition of 4.55 s, while the Edge Impulse model has an average of 3.83 s. The X-CUBE-AI tool showed that the STM model occupies 5.52 Kbytes of RAM and 7.8 Kbytes of ROM. The Edge Impulse model occupies 7 Kbytes of RAM and 38.2 Kbytes of ROM. Furthermore, the Edge Impulse platform provides automated processes for building and exporting a model, as opposed to the STM software tools, which do not provide such automated processes but give the user the flexibility to customize the process when creating a complete project.

The Edge Impulse software development platform has a time limit for the process of calculations available to the user. This is because all the processes for both the pre-processing of data and the training of the model are performed in the cloud. Contrarywise, for the development of a project using the software tools of STM, as well as for the training of the model in Python, there is no such restriction, as these procedures are performed on the user’s personal computer. Therefore, the slight difference in the performance of the inferencing of the EDGE-AI node between the two design flows is due to the selection of the CNN model, which refers to the step regarding the definition of the data processing method in the Edge Impulse methodology of Figure 2. This is a one-step process without iterations while in the case of the STM method, engineers are able to trade-off among neural network training accuracy loss and compression factor (see first iteration in the STM method of Figure 2). Edge Impulse does not provide this capability. Although different CNN architectures certainly play an important role in models’ performance differences, another contributing factor to these differences constitutes the difference in the models’ conversion procedures that STM method and Edge Impulse exhibit.

By unifying three scientific domains (i.e., audio signal processing, deep learning, and EDGE-AI), we concluded that there are two possible and efficient approaches that can be followed in order to develop a process for the operational state recognition for a DC motor via its sound with an AI model executing locally on an embedded system. The first approach prerequisites the user’s expertise in all the aforementioned three scientific domains but provide more degrees of intervention in the design cycle (i.e., the choice of using STM software tools for the deployment stage, modifications in the AI model), and the second one, with less intervention actions that needs no extensive technical expertise to be implemented (i.e., the choice of using the Edge Impulse platform). Therefore, the method selection for the specific application can be based on the aforementioned factors because both models were applicable and efficiently met the requirements of the case study that was explored in this work.

## 6. Discussion—EDGE AI Implementation vs. Cloud Computing for Predictive Maintenance

In order to evaluate the performance differences of the EDGE AI nodes over cloud computing operations, we considered two modes of operation for the STM32 Discovery Kit IoT Node. The first one constituted the EDGE AI mode and the second one was the cloud computing mode. In the EDGE AI scenario, the total amount of data to be processed by the board during inference was 8.4575 Kbytes. Initially, 16,384 bits were processed by the board to collect the audio samples needed for the calculation of one LogMel spectrogram (1024 samples × 16-bit sample resolution). Moreover, the LogMel spectrogram occupied 7680 bits (30 × 32 × 8 bits) and the neural network’s inference calculations needed 5.52 Kbytes of the board’s RAM. The latency of data processing for the extraction of a single result for EDGE AI mode was, in total, 1064.767 ms (i.e., 1024 ms for sample collection and 40.767 ms for inference extraction). Finally, the data transmitted over the network contained the result of the model, which was composed of three percentage numbers for the three classes (i.e., “good”, “broken”, and “heavy load”), as well as their class labels.

On the other hand, for the cloud computing mode, the board consumes 2 kB for data processing during inference because it only sends the 1024 PCM audio samples to the cloud. Furthermore, the latency of data processing is 1024 ms, which is slightly faster than EDGE-AI because it only refers to the time needed for the collection of audio samples. Finally, the data size transmitted over the network is 1.9844 Kbytes because they include the audio data window of 1024 ms, while for the EDGE AI mode it is only 32 bytes (the inference of the CNN network). Therefore, the conclusion regarding the comparison of the two modes, is that the main advantage of the EDGE-AI mode lies on the part of data transmission over the network. Table 11 summarizes the aforementioned evaluation.

It is deduced that the presented approach can be incorporated in various other applications where EDGE-AI is required for acoustic signal classification in real time (e.g., voice commands for robot manipulation, noise-levels in smart-city environments, etc.). Furthermore, the resource-constrained environment of MCUs has led us to develop a signal processing procedure where the acoustic signal is captured, processed, and transformed using less arithmetic precision than a PC-based SW. In this way, the sound signal has been efficiently fed to the CNN algorithm in real time and can be used as a blueprint for similar approaches.

Furthermore, supplementary model trainings are required to be performed for different DC motors. Therefore, in order to sufficiently incorporate our EDGE-AI node into an environment where various DC motors of various power capabilities that exhibit different sounds associated with their operational conditions, in comparison to the DC motor used in our case studies, it is important to re-execute the training steps for the different dataset to match the inference algorithm to the specific characteristics of the industrial environment. If the performance results are not adequate, changes in the CNN model could be enacted to cope with the different characteristics. Additionally, as mentioned in previous sections, our implemented EDGE-AI node has been trained using the sound data of [7]. The authors of this work have captured audio data based on the different levels of the voltage supply of the DC motor, since many motor faults can be caused or emulated by different voltage supply levels. Therefore, for the recognition of specific operational states of the specific DC motor where the employed data have been associated, our EDGE-AI node could perform efficiently. However, in order to efficiently identify all fault aspects of the specific DC motor, such as the short-circuited armature and field coils, the lack of ventilation, the hitting of rotating parts on the stationary parts, the worn nature of the bearings, etc., more audio data are required.

## 7. Conclusions

In this work, we presented two methodological approaches of EDGE AI nodes, used for the operational state recognition of a DC motor through sound signals, by the aspect of enhancing the efficiency and reducing valuable time regarding the identification of possible changes in the engine’s functionality. These particular aspects can be significantly beneficial in predictive maintenance operations. The two methodological approaches were developed and evaluated for the deployment of CNNs on microcontrollers and especially on the STM32 Discovery Kit IoT Node. The implementations’ design regarding the signal processing, data path, and the necessary transformations were presented in detail. It was demonstrated that both methods resulted in the efficient implementations of CNN models that can perform high accuracy and low latency classifications of the DC motor’s operational states, based on sound signals. The benefits over a relevant cloud computing implementation were also shown. Future work includes the incorporation of additional sensors that can sense vibrations and proximity to assisting the CNN models to recognize the operational states of multiple DC motors installed in an industrial environment. Finally, the efficient models’ performance on a dataset with three operational states opens the exploratory space for the problem’s augmentation with even more classes, which can include different operational states of the motor, as well as specific failure types.

## Figures and Tables

**Table 1:** Analysis of the methods used for the diagnosis of the DC motor faults.

Related Work	Monitoring Variable	Diagnosis Algorithm	Computation Unit	Cloud Support
[9]	Speed, temperature, current, and voltage	Simple Heuristic	Arduino and Local PC	No
[10]	Current	SVM, CNN, and LSTM	Local PC	No
[11]	armature winding current, resistance, input voltage and inductance, rotor angular speed, and torque constant	Output feedback approximate dynamic programming	Cloud Connectivity	Yes
[12]	Audio signals	Decision tree learning	Local PC	No
[13]	Acoustic signal	Linear discriminant analysis, NN, and NM	Local PC	No
[14]	Acoustic signal and Vibration	SVM	Local PC, microphone, and smartphone	No
[16]	Acoustic signal	NN, NM, SOM, and BNN	Local PC	No
[17]	Vibration signal	1-DCNN	Local PC	No
[18]	Acoustic signal	ANN	Local PC	No
[3]	Vibration	DNN	MCU	No
[19]	Acoustic signal of car engines	ANN, CRNN	MCU	No
This Work	Audio signal	CNN	MCU	No

**Table 2:** Audio recordings from the IDMT-ISA-ELECTRIC-ENGINE dataset.

Type of Audio Recordings	Duration (min)
Pure	15
Talking	18
Atmo (High)	9
Atmo (Medium)	9
Atmo (Low)	9
White Noise	9
Stress Test	6

**Table 3:** Confusion matrix of the Keras CNN model for the implementation on STM tools.

	Good	Broken	Heavy Load
**Good**	10,637	1	2
**Broken**	0	10,587	8
**Heavy load**	32	0	10,635

**Table 4:** Confusion matrix of the generated C-Model.

	Good	Broken	Heavy Load
**Good**	664	0	0
**Broken**	0	574	0
**Heavy load**	0	0	633

**Table 5:** Performance characteristics of the Edge Impulse model.

Metrics	Values
Accuracy (%)	97.8
Loss (%)	0.06
Peak RAM Usage (Kbytes)	7.0
ROM Usage (Kbytes)	38.2
Inference Time (ms)	16

**Table 6:** Confusion matrix of the generated Edge Impulse implementation.

	Good	Broken	Heavy Load
**Good**	504	5	4
**Broken**	4	479	0
**Heavy load**	13	7	512

**Table 7:** Average percentage indications for each class and environmental background, along with transition response times between classes for the STM’s methodology model.

	Pure	Atmo Medium	White Noise	Custom Stress Test
Good-to-Broken DC Motor State Transition				
Good class average percentage (%)	92	89	84	82
Broken class average percentage (%)	89	89	91	61
Transition response time between classes (s)	4.46	4.61	4.25	5.22
Good to Heavy Load DC Motor State Transition				
Good class average percentage (%)	90	90	89	89
Broken class average percentage (%)	87	79	84	58
Transition response time between classes (s)	5.43	4.86	4.63	9.07
Heavy Load to Broken DC Motor State Transition				
Good class average percentage (%)	89	83	78	56
Broken class average percentage (%)	89	88	89	72
Transition response time between classes (s)	3.68	4.16	3.97	4.54

**Table 8:** Average percentage indications for each class and environmental background along with transition response times between classes for the Edge Impulse methodology model.

	Pure	Atmo Medium	White Noise	Custom Stress Test
Good to Broken DC Motor State Transition				
Good class average percentage (%)	87.2	87.1	80.4	54.9
Broken class average percentage (%)	99.6	85.7	99.6	92.5
Transition response time between classes (s)	3.31	3.94	2.13	2.62
Good to Heavy Load DC Motor State Transition				
Good class average percentage (%)	95.9	95.8	96.4	72.6
Broken class average percentage (%)	99.6	99.2	99.6	84.6
Transition response time between classes (s)	2.92	5.17	3.96	5.8
Heavy Load to Broken DC Motor State Transition				
Good class average percentage (%)	99.6	99.5	97.7	84.9
Broken class average percentage (%)	99.6	85.8	99.6	68.7
Transition response time between classes (s)	3.23	5.31	3.69	2.31

**Table 9:** Confusion matrix of the STM model.

	Good	Broken	Heavy Load
**Good**	100%	0%	0%
**Broken**	5.90%	89.26%	4.84%
**Heavy load**	11.16%	0%	88.84%

**Table 10:** Confusion matrix of the Edge Impulse model.

	Good	Broken	Heavy Load
**Good**	89.06%	0%	10.94%
**Broken**	2.63%	94.74%	2.63%
**Heavy load**	3.65%	0.73%	95.62%

**Table 11:** Comparison between EDGE-AI and cloud computing modes of the STM32 Discover Kit IoT Node.

Data Processing Mode	Data Size Processed During Inference	Latency of Data Processing	Data Transmitted Over the Network
EDGE-AI	8.4575 Kbytes	1064.767 ms	32 bytes
Cloud Computing	2 Kbytes	1024 ms	1.9844 Kbytes

## Data Availability

The data presented in this study are available on request from the corresponding author.
